# Is there an association between periodontal disease and root caries? A systematic review and meta-analysis

**DOI:** 10.1371/journal.pone.0285955

**Published:** 2023-11-16

**Authors:** Rayssa Maitê Farias Nazário, Deborah Ribeiro Frazão, Beatriz Rodrigues Risuenho Peinado, Maria Karolina Martins Ferreira, Railson de Oliveira Ferreira, Marcela Baraúna Magno, Nathalia Carolina Fernandes Fagundes, Maria Tereza Campos Vidigal, Luiz Renato Paranhos, Lucianne Cople Maia, Rafael Rodrigues Lima

**Affiliations:** 1 Functional and Structural Biology Laboratory, Institute of Biological Sciences, Federal University of Pará, Belém, Brazil; 2 Department of Pediatric Dentistry and Orthodontics, Faculty of Dentistry, Federal University of Rio de Janeiro, Rio de Janeiro, Brazil; 3 Faculty of Dentistry, Faculty of Medicine and Dentistry, University of Alberta, Edmonton, Canada; 4 Department of Preventive and Social Dentistry, Faculty of Dentistry, Federal University of Uberlândia, Uberlândia, Minas Gerais, Brazil; Universidad Privada San Juan Bautista, PERU

## Abstract

Some periodontal diseases can be associated with cariogenic bacterial growth due to various oral health imbalances. This fact may be linked to a greater development of root caries. Thus, this systematic review analyzed the evidence on the association between periodontal disease and root caries. An electronic search was performed in five databases (Cochrane Library, LILACS, MedLine *via* PubMed, Scopus, and Web of Science) and two additional sources (Google Scholar and Open Grey) to partially capture the grey literature. The PECO strategy was used to identify prospective or retrospective observational studies assessing root caries in patients with periodontal disease without language or year publication restrictions. Two reviewers extracted data and evaluated the individual risk of bias in the eligible studies. Random effects meta-analyses were performed to calculate the Odds Ratio (OR). The risk of bias was assessed by the NIH tool, and the certainty of evidence was classified according to the GRADE tool. There were 1,725 studies retrieved, of which four met the eligibility criteria. All of them were evaluated for the control statements for possible confounders, bias consideration, and confounding factors because they had multivariate analysis. Adults with periodontal disease had a greater chance of presenting root caries than adults without, with OR 1.38 [CI 1.25, 1.53]. The certainty of evidence was classified as very low. Within the limits presented in this review, there was an association between periodontal disease and root caries, highlighted in the qualitative synthesis and the meta-analysis results.

## Introduction

Periodontal health is the absence of histological signs of periodontal inflammation and the absence of signs of periodontium anatomical change [1, 2]. Periodontitis, in turn, is one of the types of periodontal disease, and it is the most critical public health problem due to its widespread prevalence [3]. Periodontitis is a chronic multifactorial inflammatory illness characterized by increasing loss of the tooth-supporting system and associated with dysbiosis plaque biofilms [4]. The primary characteristics of this disease include gingival bleeding, radiographic alveolar bone loss, periodontal pocketing, and clinical attachment loss (CAL), which is the loss of periodontal tissue support [5]. Periodontal disease may impair quality of life, mastication, esthetics, and self-confidence [6, 7].

Any carious lesion that develops on the tooth’s root surface is called root caries [8]. It is described as a cavitated or non-cavitated lesion that is not connected to the neighboring enamel and is located below the cementoenamel junction (CEJ) [8, 9]. Cementum and underlying dentine are both affected by root caries, which is discolored, mushy, and poorly defined [8]. Upon progression, root caries may potentially lead to dental pulp necrosis and the destruction of the surrounding periodontal tissues [9].

Root caries has a bidirectional effect on periodontal tissues and tooth surfaces, which worsens the impact of both illnesses on a patient’s oral health [10]. Usually, gingival tissues cover the root surface, but when the periodontal disease develops, and gingival recession occurs, the microbiome of the exposed root may change from anaerobic to aerobic, which could speed up the demineralization process in the presence of additional risk factors [11–13].

Metabolically specialized microorganisms cause caries and periodontal disease. Positive feedback loops are involved in the progression of these diseases, but they are triggered by different stressors. In caries, continued exposure to sugars and organic acid fermentation can result in a positive feedback loop, increasing the amount of acidogenic and aciduric microorganisms and raising the pH of the medium. Plaque accumulation causes inflammation in gingivitis, and a positive feedback cycle increases inflammatory factors, which can dysregulate the host immune response and destroy periodontal tissues [4, 14].

Moreover, root caries is challenging to identify, especially in the early stages of demineralization [15]. However, it is a consensus that a visual and tactile inspection of the dry tooth surface can detect and measure the severity of root caries through its specific evaluation index [16]. Furthermore, with aging, the presence of gingival recession can occur as a sequel to periodontitis, resulting in occurrence at the same time of caries and periodontal disease, usually non-communicable diseases [17]. Thus, a negative mutualism between both diseases, also associated with other aging conditions, may indicate a risk of demineralization of the root surface due to the anatomical discrepancies present on this surface, which was once protected by periodontal support tissues [17].

Therefore, since there is some evidence regarding the relationship between periodontal disease and root caries, this systematic review aims to summarize the findings on whether there is an association between periodontal disease and root caries in adult patients.

## Materials and methods

### Protocol registration

The protocol was reported according to the Preferred Reporting Items for Systematic Review and Meta-Analysis Protocols (PRISMA-P) [18] and registered in the Open Science Framework (OSF) database under the DOI: https://doi.org/10.17605/OSF.IO/R97HY. This systematic review was conducted according to the Preferred Reporting Items for Systematic Reviews and Meta-Analyses (PRISMA) [19], the Joanna Briggs Institute (JBI) Manual [20], and to the Conducting Systematic Reviews and Meta-Analyses of Observational Studies of Etiology (COSMOS-E) guideline [21].

### Research question and eligibility criteria

The review was designed to answer the following question: “Is there any association between periodontal disease and root caries?” following the PECO strategy for structuring, in which: P (population), E (exposure), C (comparison), and O (outcome).

#### Inclusion criteria

- Population: adult patients;
- Exposure: subjects with periodontal disease;
- Comparator: subjects without periodontal disease;
- Outcome: root caries.
- Study design: prospective or retrospective observational studies (cross-sectional, case-control, or cohort studies).

There were no restrictions on publication language or year.

#### Exclusion criteria

- Studies with sample overlapping (in this case, considering the most recent study that best described the methodology and results);
- Studies presenting a qualitative inquiry, studies of intervention, case reports, case series, literature reviews, editorials, letters to the editor, personal opinions, books, and book chapters.

### Sources of information, search, and selection of studies

The electronic searches were performed in the Cochrane Library, Latin American and Caribbean Health Science Literature (LILACS), Medline (*via* PubMed), Scopus, and Web of Science. Google Scholar and Open Grey were used to partially capture grey literature. These steps were performed to minimize the selection bias. Search updates in all databases were performed until April 2023. The search descriptors were selected according to the MeSH (Medical Subject Headings), DeCS (Health Sciences Descriptors), and Emtree (Embase Subject Headings) resources. The main descriptors used to compose the search strategies were: Root Caries, Periodontal Disease, Periodontitis, Gingival Disease, Adult. Several combinations among the descriptors were performed with the Boolean operators “AND” and “OR”, respecting the syntax rules of each database (S1 Table in S1 File).

The results were exported to a reference software manager (EndNote X9™, Clarivate™ Analytics, Philadelphia, USA). Duplicates were firstly removed using the automated software tool, and then manually checked. The grey literature was manually analyzed, simultaneously and fully, with Microsoft Word™ 2010 (Microsoft™ Ltd., Washington, USA).

Before selecting the studies, two reviewers performed a calibration exercise in which they discussed the eligibility criteria and applied them to a sample of 20% of the studies retrieved to determine inter-examiner agreement. After reaching an adequate level of agreement (Kappa ≥ 0.81), the selection started (Table 1). In the first phase, two eligibility reviewers (RMFN and BRRP) independently analyzed the titles and abstracts of the studies. A third examiner (DRF) interpreted and defined disagreements between the examiners. In the second phase, the full texts of the preliminary eligible studies were assessed.

**Table 1:** Symmetric measures of agreement between variables with kappa test.

Symmetric Measures					
		Value	Asymptotic Standard Error^a^	Approximate T^b^	Approximate Significance
Measure of Agreement	Kappa	,887	,112	15,466	<,001
N of Valid Cases		300			

a. Not assuming the null hypothesis.

b. Using the asymptotic standard error assuming the null hypothesis

### Data collection

Before data extraction, a calibration exercise was performed to ensure consistency between the reviewers, in which the data from three eligible studies were extracted jointly. After the calibration, two reviewers (RMFN and BRRP) independently and blindly extracted the data from the eligible studies. In cases of disagreement about data extraction, a third reviewer (DRF) was consulted.

The following data were extracted from the articles: study identification (author, year, country, location, and application of ethical criterion), sample characteristics (number of patients with and without root caries, distribution by sex and average age), data collection and processing characteristics (root caries diagnostic method, periodontal assessment method, and type of statistical analysis used), main results (main outcomes from each study, number of patients with periodontal disease diagnosed with root caries). In case of incomplete or insufficient data, the corresponding authors were contacted via e-mail up to three times, with weekly intervals.

### Risk of bias assessment

#### Evaluation of methodological quality

Included studies were assessed for the risk of individual bias with the National Institutes of Health (NIH) Quality Assessment Tool for Observational Cohort and Cross-Sectional Studies [22]. Two authors (DRF and MKMF) assessed each domain independently for the quality evaluation and risk of bias, as recommended by the PRISMA statement [19]. A third examiner (ROF) interpreted and defined disagreements between the examiners. The methodological quality of the studies was assessed using checklist criteria. Each study’s quality was rated as poor, fair, or good after answering a set of 14 questions.

#### Evaluation of control statements for possible confounders and bias consideration

This evaluation was based on a previous study of Hemkens et al., 2018 [23]. All eligible studies were initially analyzed regarding the explicit mention of multivariate analysis, an essential statistical test to control possible confounders. Studies that did not perform multivariate analysis were excluded. Moreover, the remaining studies were critically appraised by two independent reviewers (MTCV and LRP), whose disagreements were solved by a third reviewer (RRL). The abstract and Discussion sections of each eligible study were read and evaluated by six questions. The last question considered the Conclusion section. If there was no specific section for the Conclusion, the last paragraph of the Discussion section was assessed instead.

#### Assessment of confounding factors

This assessment was based on a previous study of Wallach et al., 2020 [24]. The critically appraised studies had their Methods and Results sections read and assessed by two independent reviewers (MTCV and LRP), and a third reviewer (RRL) decided their conflicts. This step allowed the identification of variables and confounding domains presented in each study. The variables were also classified as adjustment (used in multivariate analysis or Poisson’s regression to control possible confounders), stratification (used in sample selection to make strata) or matching variables (used to pair known characteristics between study participants or groups).

### Data synthesis and meta-analysis

The data collected from the studies selected were organized in Microsoft Excel™ 2019 spreadsheets (Microsoft™ Ltd., Washington, USA) and described narratively (qualitative synthesis). Quantitative data were analyzed using RevMan software (Review Manager 5.3, Copenhagen, Denmark) to evaluate the association between periodontal disease and root caries. Standardization of evaluations was considered, and only studies with low risk of bias that report the association between CALoss (≤3mm and ≥4mm) and root caries (diagnosed with established indexes) were included.

The odds ratio (OR) and its 95% CI of the association between these diseases (periodontal disease and root caries) from included studies were extracted. The adjusted odds ratio was also used whenever possible; otherwise, crude odds ratio estimates were considered to measure the effect as a log OR and the standard error of the log OR using generic inverse-variance weighting method. When necessary, the effect estimates were converted to OR with the help of RevMan software tools.

The fixed effect model was applied when an extremely low number of studies were included (three or fewer studies), and the random effect was applied when four or more studies were included in the meta-analysis [25]. Heterogeneity was tested using the I^2^ index and the predictor interval were calculated if random effect was applied [26].

#### Certainty of evidence

The certainty of the evidence and the strength of recommendations were evaluated using the Grade of Recommendation, Assessment, Development, and Evaluation (GRADE) tool. The GRADEpro GDT software (http://gdt.guidelinedevelopment.org) was used to summarize the results. The evaluation for downgrade was based on the risk of bias, inconsistency, indirectness, imprecision, and publication bias. The assessment to increase certainty was based on large magnitude of effect, dose response, and confounders likely minimize the effect. The certainty of evidence would be rated as low or very low due to the design of the included studies [27].

## Results

### Study selection

**Fig 1:**
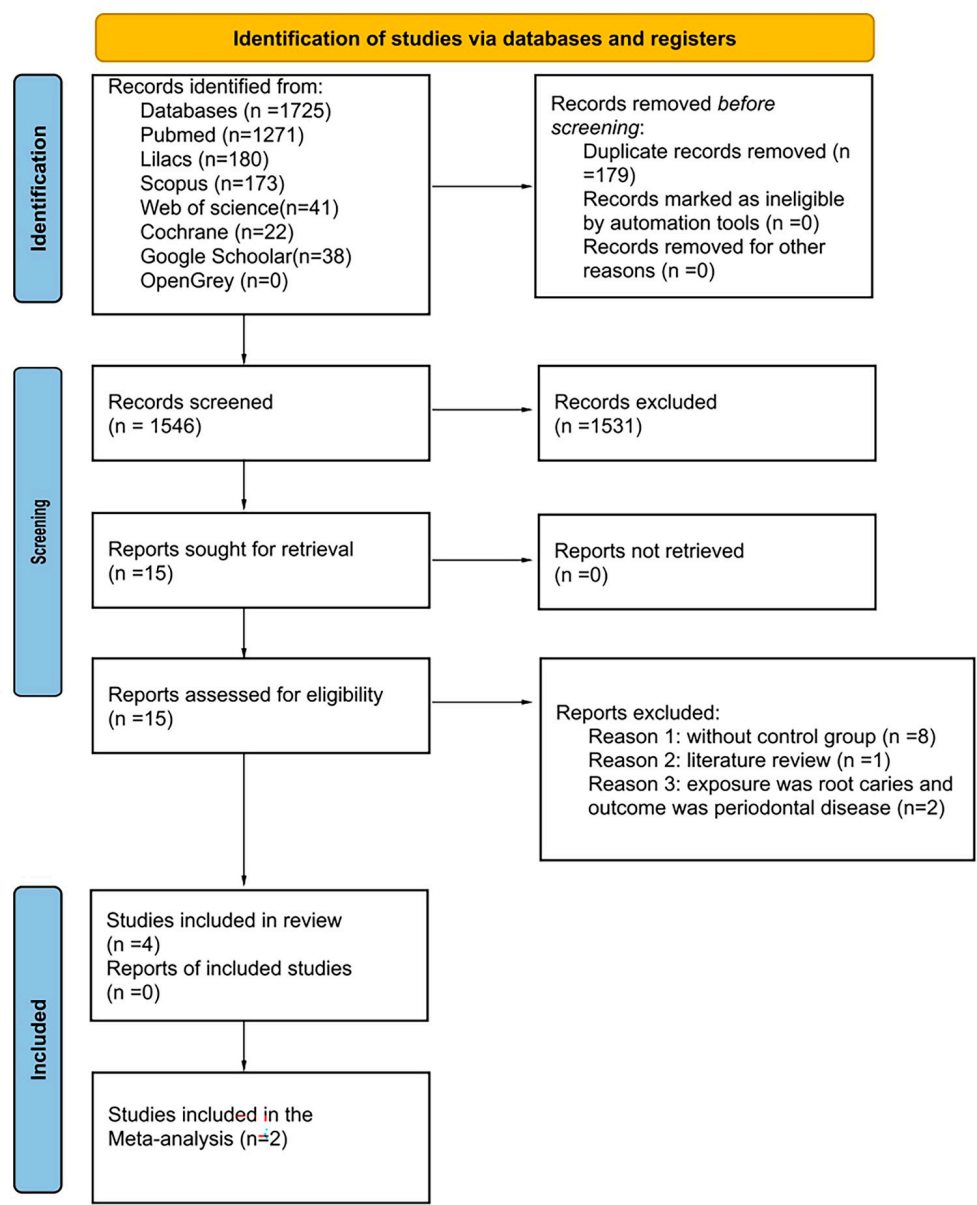
Flow diagram of databases searched according to PRISMA guidelines (Preferred reporting items for systematic review and meta-analysis).

Initially, 1,725 records were identified from the seven electronic databases, including grey literature sources. After removing the duplicates, 1,546 results remained for the analysis. A careful reading of the titles and abstracts resulted in the exclusion of 1,531 studies, leaving 15 for full-text reading. After that, 11 articles were excluded after full-text analysis: eight studies did not have a control group [28–35], two studies described root caries as exposure, not outcome [36, 37], and one was a literature review [17]. Therefore, four articles were included in the qualitative synthesis [10, 38–40]. Fig 1 displays details of the study selection process.

### Study characteristics

The studies were carried out in different countries, including China [40] England [10] Japan [39] and Finland [38]. Adding the total number of study participants, the result was 18724 people. The mean age across the studies was about 53 years.

All studies assessed periodontal diseases through clinical examinations that contributed to a periodontitis diagnosis. Among the clinical parameters, three studies evaluated CAL (Clinical Attachment Loss) [10, 39, 40], 2 verified it through Pocket Deep (PD) [10, 39], one observed plaque level and Gingival inflammation [38], and one verified Basic periodontal examination (BPE) [10].

Regarding root caries analysis, 1 study searched Root caries index (RCI) [38], 2 investigated Decayed, Missing and Filled Teeth (DMFT) [10, 40], and 1 performed several analyzes such as numbers of root surface decayed teeth (RDT), root surface filled teeth (RFT), root surface decayed and filled teeth (RDFT) [39]. All studies’ characteristics are presented in Table 2.

**Table 2:** Summary of the individual studies’ characteristics and results.

Author, year (country)	Participants			Periodontal disease evaluation indexes	Root caries assessment	Statistical analysis	Results	Conclusion
	Source of sample	Sample size	Age					
Yu et al., 2021 (China)	4th National Oral Health Survey	8841	≥35 years	Clinical Attachment Loss (CAL)	Decayed and Filled Teeth (DFT)	Chi-square test and Logistic regression	**Middle-aged patients (35–44 years)** **Root caries, n (%; 95% CI)** Without periodontal disease (CAL≤3 mm): 62 (2.1; 1.6–2.7) With periodontal disease (CAL = 4–5 mm): 40 (3.6; 2.6–4.8) With periodontal disease (CAL≥6 mm): 25 (7.4; 4.8–10.7) (p<0.001) **Middle-aged patients (65–74 years)** **Root caries, n (%; 95% CI)** Without periodontal disease (CAL≤3 mm): 70 (8.4; 6.6–10.5) With periodontal disease (CAL = 4–5 mm): 231 (16.2; 14.3–18.2) With periodontal disease (CAL≥6 mm): 407 (21.9; 20.0–23.8) (p<0.001) **Middle-aged patients (35–44 years)** **Odds Ratio (95% CI)** Model 1^a^: 1.43 (1.27–1.59) * Model 2^b^: 1.44 (1.28–1.6) * Model 3^c^: 1.40 (1.24–1.56) * *: p<0.001 **Middle-aged patients (35–44 years)** **Odds Ratio (95% CI)** Model 1^a^: 1.26 (1.19–1.33) * Model 2^b^: 1.28 (1.21–1.35) * Model 3^c^: 1.28 (1.21–1.35) * *: p<0.001	The authors showed that middle-aged and elderly people presented root caries positively correlated with periodontitis.
AlQobaly et al., 2020 (England)	Adult Dental Health Survey (ADHS) 2009	4738	≥35 years	Basic periodontal examination (BPE) Pocket Depth (PD) Clinical attachment level (CAL)	Decayed, Missing and Filled Teeth (DMFT). Root caries was only indicated by the sum of decayed and filled roots.	Negative binomial regression	**Mean root caries (95% CI)** Without periodontal disease: 7.65 (7.32‐7.98) With periodontal disease: 10.04 (9.73‐10.36) **Rate Ratio (RR):** Without periodontal disease: Reference With periodontal disease: 1.23 95% CI: 1.16–1.30 (p < 0.001)	This study shows that individuals with periodontitis had 23% higher risk of presenting root caries than subjects without periodontitis.
Saotome et al., 2006 (Japan)	Residents of Niigata City, Japan	368	75 years	Probing pocket depth (pPD) Clinical attachment level (CAL) Average pocket depth (aPD)	Numbers of root surface decayed teeth (RDT) Root surface filled teeth (RFT) Root surface decayed and filled teeth (RDFT)	Chi-square test Mann–Whitney U-test	The presence of root caries was examined in 356 subjects with gingival recession. The control group had a healthy periodontal status, and the exposed group had a rate of sites with >4 mm of attachment loss and a rate of sites with >4 mm of pocket depth. Untreated caries on root surfaces were found in 23.0% of those individuals	These results indicate that attachment loss is associated with the number of root caries.
Vehkalahti and Paunio, 1994 (Finland)	Finnish population	4777	≥30 years	Gingival inflammation subgingival plaque retention	Root caries index (RCI)	The method used was to fit a logit model to the data using a Glim3 computer program.	**Odds Ratio**^**#**^ Women: Gingival inflammation: 3.4* Pockets 4–6 mm: 2.9* Pockets > 6 mm: 3.2* Men: Gingival inflammation: 5.3* Pockets 4–6 mm: 5.4* Pockets > 6 mm: 9.3* ^#^: compared to subjects with healthy periodontum *: p<0.001	This study showed an association between root caries occurrence and a subject’s periodontal state.

• CI: Confidence Interval

• ^a^: DFT was included as the only independent variable in the ordered logistic regression analysis

• ^b^: Social economic status, sex, area, education level, and household income per capita were added to Model 1

• ^c^: Oral health-related behaviours such as smoking status, tooth brushing frequency, use of dental floss, use of a toothpick, alcohol consumption and diabetes were added to Model 2

### Results of individual studies and syntheses

All studies evaluated the association between the pathologies through regression and correlation. For instance, through using binomial regression, AlQobaly and Sabbah (2020) [10] observed in their study, with a population of 4738, that people with periodontitis (PD or LoA ≥ 4 mm) had a significant correlation with root DMFT (RR: 1,03, CI 95%: 1,01‐1,05).

The correlation of periodontal status with untreated root surfaces caries was explored in a study involving 368 people by Saotome et al. (2006) [39]. Multiple regression analysis revealed a correlation between average attachment loss aAL (p = 0.014) and attachment loss greater than 4 mm rAL4 (p = 0.086). Furthermore, a multiple regression analysis the periodontal status aPD (p = 0.113) and pocket depth greater than 4mm rPD4 (p = 0.532).

Vehkalahti and Paunio (1994) [38] found an association between the diseases in a study of 4777 individuals. Then, women with gingival inflammation presented 3.4 more chance of having root caries than healthy women. Finally, Yu et al. (2021) [40] defined the relationship between root caries and periodontal disease using ordinal logistic regression models. They discovered a significant association between the diseases, with an OR of 1.40 and a 95 percent confidence interval of 1.24–1.56.

According to Yu et al. (2021) study [40], sex is correlated to simultaneous root caries and severe periodontal disease. There were 54.4% of women and 45.6% of men who had CAL ≤ 3 mm. It was found that 45.1% of men and 54.9% of women had CAL ≤ 4–5 mm. Only 31% of women and 69% of men had CAL ≤ 6mm. These results indicate that men have a higher susceptibility to have both diseases simultaneously. In Alqobaly et al. (2020) article [10], DMFT was higher among females, whereas root caries was higher among males. Also, men had a 9.61 chance of having root caries and women had an 8.29 chance. According to the findings of Vehkalahti et al. (1994) [38], subgingival plaque retention was associated with a higher risk of root caries among women (odds ratio 2.5) than it was among men (odds ratio 1.4). For women, the odds ratio for developing root caries was 2.3, while for men it was 4.9.

### Risk of bias assessment

#### Methodological quality of the eligible studies

Three studies presented a low risk of bias [10, 38, 40] because they met all the criteria from the checklist. However, the other study demonstrated a fair quality because they have not clearly defined inclusion criteria in the sample, have not identified confounding factors, and have not described how to deal with it [39]. Table 3 shows more details about this methodological evaluation of eligible studies.

**Table 3:** Quality assessment tool for observational cohort and cross-sectional studies.

CRITERIA	YU ET AL., 2021	ALQOBALY ET AL., 2020	SAOTOME ET AL., 2006	VEHKALAHTI, ET AL., 1994
**1. Was the research question or objective in this paper clearly stated?**	Yes	Yes	Yes	Yes
**2. Was the study population clearly specified and defined?**	Yes	Yes	Yes	Yes
**3. Was the participation rate of eligible persons at least 50%?**	Yes	Yes	Yes	Yes
**4. Were all the subjects selected or recruited from the same or similar populations (including the same time period)? Were inclusion and exclusion criteria for being in the study prespecified and applied uniformly to all participants?**	Yes	No	Yes	No
**5. Was a sample size justification, power description, or variance and effect estimates provided?**	Yes	Yes	No	Yes
**6. For the analyses in this paper, were the exposure(s) of interest measured prior to the outcome(s) being measured?**	Yes	Yes	Yes	Yes
**7. Was the timeframe sufficient so that one could reasonably expect to see an association between exposure and outcome if it existed?**	Yes	Yes	Yes	Yes
**8. For exposures that can vary in amount or level, did the study examine different levels of the exposure as related to the outcome (e.g., categories of exposure, or exposure measured as continuous variable)?**	Yes	Yes	Yes	Yes
**9. Were the exposure measures (independent variables) clearly defined, valid, reliable, and implemented consistently across all study participants?**	Yes	Yes	Yes	Yes
**10. Was the exposure(s) assessed more than once over time?**	No	No	No	No
**11. Were the outcome measures (dependent variables) clearly defined, valid, reliable, and implemented consistently across all study participants?**	Yes	Yes	Yes	Yes
**12. Were the outcome assessors blinded to the exposure status of participants?**	No	No	No	No
**13. Was loss to follow-up after baseline 20% or less?**	Na	Na	Na	Na
**14. Were key potential confounding variables measured and adjusted statistically for their impact on the relationship between exposure(s) and outcome(s)?**	Yes	Yes	No	Yes

CD, cannot determine; NA, not applicable; NR, not reported.

#### Evaluation of control statements for possible confounders and bias consideration

All eligible studies had performed multivariate analysis and were selected to be critically appraised. Two studies [38, 40] made a specific mention of the term “confounding.” Two studies [10, 38]) used the term “bias.” Two studies [38, 40] mentioned non-adjusted variables as not measured: the number of periodontally diseased sites [38] and missing teeth due to caries [40]. Two studies [10, 38] mentioned their results as possibly being affected by confounders, and one study [40] mentioned it as probably. Only one study [38] stated the need for caution in interpreting their results. Only one study [10] included limitations regarding confounders in their conclusions. The results of the evaluation of control statements for possible confounders and bias consideration are summarized in Table 4.

**Table 4:** Evaluation of control statements for possible confounders and bias consideration.

Section	Question	Possible answers with explanation	N (%)
Abstract and Discussion	Is the term “confounding” mentioned in Abstract or Discussion?	**Specific:** if authors used the exact term “confounding”.	2 (50%)
		**Alluded:** if authors used a similar term or phrase.	2 (50%)
		**No:** if the authors used neither the exact nor similar term.	0
	Is the term “bias” used in Abstract or Discussion?	**Yes:** if authors used the term “bias”.	2 (50%)
		**No:** if authors did not use this term.	2 (50%)
	Is any specific mention about non-adjusted variables in Abstract or Discussion?	**Yes:** if there was specific mention about non-adjusted variables with no reasons presented.	0
		**Not measured:** if there was specific mention about non-adjusted variable not being measured.	2 (50%)
		**Other reasons:** if there was specific mention about non-adjust variables and with plausible reasons for not adjusting them.	0
		**No reasons:** if there was specific mention about non-adjusted variables and with implausible reasons for not adjusting them.	0
		**No:** if there was no mention about any non-adjusted variable.	2 (50%)
	Is there any mention about confounders affecting results in Abstract or Discussion?	**Likely:** if authors used terms such as “likely” or convincing statements that confounders were not controlled.	1 (25%)
		**Possibly:** if authors used terms such as “possibly” or unsure statements that confounders were or were not controlled.	2 (50%)
		**Unlikely:** if authors used terms such as “unlikely” or convincing statements that confounders were controlled.	0
		**No mention:** if there was no mention about this possibility.	1 (25%)
	Is there any statement about the need for caution in interpretating the results?	**Yes:** if there was explicit mention about the need for caution in interpretating the results obtained in the study.	1 (25%)
		**No mention:** if there was no mention about this need for caution.	3 (75%)
Conclusion	Does Conclusion include any limitation about confounders?	**Yes:** if there was a mention about this limitation.	1 (25%)
		**No:** if there was no mention about this limitation.	3 (75%)

#### Assessment of confounding factors

A total of 56 variables were identified in selected studies. There were 41 variables used in multivariate analysis to control possible confounders. Three studies [38–40] performed sample stratification utilizing 11 variables. Only one study [39] used a matching variable, “age.”

Seven confounding domains were identified in the selected studies: (1) sociodemographic and socioeconomic; (2) behaviors; (3) dental; (4) periodontal; (5) microbiological; (6) salivary; and (7) comorbidities. The confounding domains identified in each study are presented in Table 5.

**Table 5:** Confounding domains identified in selected studies.

Author, year	Confounding domains							
	Sociodemographic and socioeconomic	Behaviors	Dental	Periodontal	Microbiological	Salivary	Comorbidities	
Yu et al., 2021	x	x	x	x	--	--		x
AlQobaly and Sabbah, 2020	x	x	x	x	--	--		--
Saotome et al., 2006	x	x	x	x	x	x		--
Vehkalahti and Paunio, 1994	x	--	x	x	--	--		--

‘x’—identified in the study; ‘--’—not identified in the study.

The sociodemographic and socioeconomic domain was the most explored, with a total of 23 variables. In contrast, salivary and comorbidities domains were the least explored with one variable in each: stimulated saliva flow and diabetes, respectively. Their description and examples of variables identified are shown in S2 Table in S1 File.

### Data synthesis and meta-analysis

Only two studies [10, 40] were included in the meta-analysis. Saotome et al. (2006) [39] and Vehkalahti and Paunio (1994) [38] did not standardize the data. Satome et al. (2006) report root caries though surface index and Vehkalahti and Paunio (1994) evaluated periodontal disease through gingival inflammation and subgingival plaque retention.

**Fig 2:**
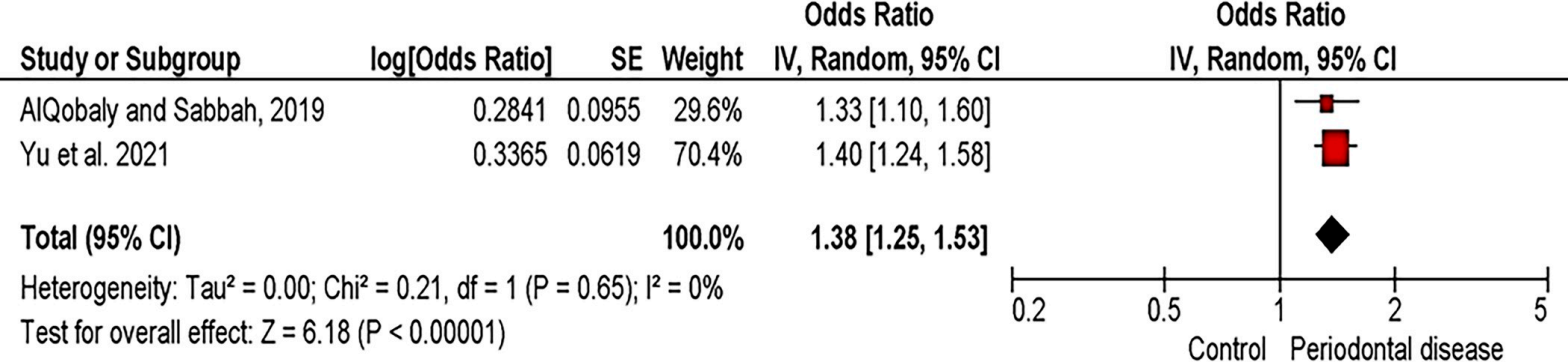
Forest plot of the association between root caries and periodontal disease (CALoss).

Adults with root caries (n = 3677) had a greater chance to present periodontal disease than control adults (without root caries, n = 3987) OR 1.38 [1.25 to 1.53] p<0.001, I^2^ = 0% (Fig 2).

### Certainty of evidence

The outcome analyzed by both studies included in the meta-analysis showed low certainty of evidence. The assessment of the certainty of evidence summary is presented in Table 6.

**Table 6:** Certainty of evidence assessed by GRADE tool.

Certainty assessment						№ of patients		Effect	Certainty
№ of studies Study design	Risk of bias	Inconsistency	Indirectness	Imprecision	Other considerations	Periodontal disease	Control	Relative (95% CI)	
Root caries									
2 observational studies	not serious	not serious	not serious	not serious	none	3677	3987	OR 1.38 (1.25 to 1.53)	⨁⨁◯◯ Low

CI: confidence interval; OR: odds ratio.

## Discussion

This systematic review assessed the association between periodontal disease and root caries. The presence of association is demonstrated by the qualitative synthesis and the meta-analysis, which found that those with periodontal disease had a 38% more chance of presenting root caries. Furthermore, the certainty of the evidence analysis indicates a low level of evidence, which is expected for observational studies. Although the studies used correlation analysis, the results did not include covariates or indicate which teeth groups were most affected.

The relevance of investigating the association between periodontal disease and root caries is because some periodontal diseases, such as periodontitis, can lead to the occurrence of gingival recession and exposure of root surfaces to the aerobic microbiota of the mouth, which may favor cariogenic bacterial growth [10]. Because root surfaces are more susceptible to caries than the enamel on coronal surfaces, this phenomenon can be used to explain the role of periodontitis in the development of root caries [41]. Moreover, periodontal disease and caries have some disease-modifying factors in common, such as age, brushing frequency, flossing, socioeconomic status, eating habits, smoking status, and others [42, 43].

Periodontal disease and root caries have been shown in the literature to have a positive relationship with age [41, 44]. Thus, it is essential to emphasize that the most prevalent periodontal disease in the elderly is periodontitis, due to biofilm accumulation mainly in the teeth’ cervical region that decreases the quality of supporting tissues [45]. Considering the lower degree of mineralization of cementum in relation to enamel in old age, coupled with the accumulation of subgingival biofilm, there is a significant possibility of the occurrence of root caries when there is dysbiosis of oral microorganisms [45].

These observations are in accordance with the results of AlQobaly and Sabbah (2020) [10], which found that root caries was more prevalent among the 55 and older age group. Moreover, according to binomial regression, the study found that adults aged 35–44 years had a 34% risk of presenting an association between root caries and periodontal disease, while adults aged 55 years or older had a 62% chance. The article by Saotome, et al. (2006) [39] investigated individuals with 75 years. They identified a positive correlation between root caries and average attachment loss, rate of sites with >4 mm of attachment loss, and rate of sites with >4 mm of pocket depth.

Another modifying factor of root caries and periodontal disease is oral hygiene habits, which, if maintained, would reduce the occurrence of both diseases. Yu et al. (2021) [40] used oral health-related behaviors as a covariate, considering the frequency of tooth brushing and flossing. Patients who did not maintain these habits had a clinical attachment loss of 4-6mm or more. This analysis was associated with root caries indirectly because patients with these clinical attachment loss values had a higher rate of root caries than patients with CAL less than 4mm [40]. Furthermore, AlQobaly and Sabbah (2020) [10] discovered a link between root caries and periodontal disease in patients who brushed their teeth only once a day or did not brush at all. Saotome et al. (2006) [39], on the other hand, confirmed the relationship between tooth brushing and flossing only in the presence of root caries, and Vehkkalahti and Paunio (1994) [38] did not investigate whether oral hygiene influenced the association between caries and root disease.

It is important to note that all articles included in the systematic review cannot address causality because they are cross-sectional studies. However, it is well known that poor oral hygiene is one of the multifactorial causes of both periodontal disease and root caries [42, 43]. Given the advanced age of the researched patients and the poor oral hygiene observed in the studies, it emphasizes the need of oral health prevention for the elderly, mainly because they are predisposed to acquire systemic disorders [46].

Although all studies assessed the association between periodontal disease and root caries by correlation or regression, only two used confounding variables to adjust the results [10, 38]. Interestingly, the study by Vehkalahti and Paunio (1994) was not included in the meta-analysis, just like Saotome et al. (2006) research. One of the reasons is that, since they were published more than fifteen years ago, the groups and disease analyses were not standardized concerning caries and periodontal disease diagnosis. As a result, the methodologies of these studies varied significantly.

The most recent periodontitis classification is based on severity levels defined by clinical interdental loss, radiographic bone loss and tooth loss, complexity, extent, and distribution [5]. Regarding root caries, Ismail et al. (2015) [47] projected a classification indicated by codes ranging from 0 to 2, considering the presence of cavitation and lesion activity. A discolored area on the root surface could be used to determine whether or not the root caries lesion is active [47]. For instance, the research from Yu et al. (2021) and AlQobaly and Sabbah (2020) examined periodontal disease considering the updated disease classification. In terms of root caries, they did not adhere to the ICCMS protocol but instead employed the same clinical evaluation as Decayed and Filled Teeth (DFT). Due to this standardization, both were included in the meta-analysis.

There was a high proportion of adjustment variables (41/56), indicating a very good statistical control of identified confounders. The multivariate analysis considers multiple known confounders that can statistically reinforce the results’ validity of observational studies [48]. However, it does not compensate for the unexplored confounding domains and does not erase the imbalance of techniques in sample selection.

Regarding the methodology quality, the only fair quality study [39] had risk of bias for its sample selection and possible confounders, respectively due to lack of inclusion criteria and lack of proper confounders’ acknowledgment. Although Saotome et al., 2006 [39] considered more confounding domains than the other eligible studies, the possible confounders within each domain and bias consideration were not adequately recognized as shown in Table 3. This represents a limitation of the study, because the data interpretation may be carelessly overestimated.

As for the most explored confounding domains, some eligible studies have shown interesting positive results for participants who were men [10, 38, 40] and older than 55 years old [10], as well as the ones with poor oral hygiene habits [10, 38, 39]. These variables may be connected by sociocultural reasons, such as women generally being more interested and active regarding their health-related education and habits, but not in an exclusive way, so both men and women can present poor oral hygiene habits and, therefore, periodontitis and root caries. Also, age is frequently related to various comorbidities, physical or coordination limitation, and changes in the daily routine habits, which may impact elderly patients’ oral hygiene and lead to the development of oral diseases. However, these cause-and-effect theories are not possible to be completely assumed based on the cross-sectional studies of this systematic review.

Detailing the least explored domains, Saotome et al., 2006 [39] uncovered two important confounding domains that were not considered in any other eligible study and are consecrated as related to caries multifactorial etiology: the microbiological and salivary domains. As stated before, it does not compensate the flaws of the study, but it is essential to state that the results are not invalid or negligible and highlight variables to be considered in further studies. Also nearly unexplored, the comorbidities domain acknowledged only by Yu et al., 2021 [40] has a valuable confounder to be studied and it is widely related to periodontal disease: diabetes. Therefore, future studies should be designed considering the known confounders and also the underestimated ones to better understand this association between periodontal disease and root caries.

The level of evidence remained low because there was no representativeness of participants in the included studies and, therefore, a low weight. Still, it is recommended that more attention be provided to individuals who present both root caries and periodontal disease, especially elderly patients. It is suggested that more preventive activities be carried out in the middle-aged to the older population and also that clinical trials be conducted to see if early treatment of root caries prevents the onset of periodontal disease; or whether periodontal therapy would minimize the appearance of root caries since both are associated, with no causative factor involved.

Therefore, some strengths of this study should be highlighted. This systematic review is a pioneer in approaching this theme. In addition, it followed strict criteria, always in accordance with the most current specific guidelines, and carried out a comprehensive individual critical analysis of eligible studies.

Nevertheless, some relevant limitations were found in the eligible studies. Due to their observational study design, they do not allow causality inference [21], not permitting identifying which pathology would be most influential in developing the other. In addition, of the four selected studies, only one was carried out in older patients and the other three in a very wide age range. This is another limitation of this systematic review, since age may be an important modulator of health status and studies that better investigate this association at different ages are still necessary. In general, few confounders were acknowledged, and three identified domains were poorly explored by selected studies. This lack of proper acknowledgment of possible confounders is in support with other studies [23]. However, it should be improved in further studies because it represents a very high possibility of unknown or underestimated confounders to be affecting the results.

In conclusion, our study provides evidence of an association between root caries and periodontal disease. However, we acknowledge that this topic is complex and multifaceted, and there are varying perspectives and among researchers and clinicians regarding the relationship between these two conditions. Some may argue that the observed association is confounded by common risk factors, such as age, diet, and oral hygiene, or that the studies included in our analysis were subject to biases and limitations. Others may suggest alternative explanations or mechanisms for the observed association, such as microbial interactions or host immune response. We therefore encourage readers to consider these different perspectives and draw their own conclusions based on the available evidence. Further research is needed to elucidate the underlying mechanisms and establish a temporal relationship between the events of this association, as well as to explore potential preventive and therapeutic strategies for these prevalent oral diseases.

## Conclusions

Within limits presented in this review, it is concluded that there was a positive association between periodontal disease and root caries, which was highlighted in the qualitative synthesis and in the result of the meta-analysis.

## Supporting information

S1 Checklist PRISMA 2020 checklist. (DOCX)

S1 File (DOCX)
