# Supplementary material for: Is there an association between periodontal disease and root caries? A systematic review and meta-analysis
Source: PLoS One. 2023 Nov 16;18(11):e0285955. doi: 10.1371/journal.pone.0285955 (PMC10653397; doi:10.1371/journal.pone.0285955)
Supplement: S1 File — (DOCX) [file pone.0285955.s002.docx]

Supplementary Material

Supplementary Table 1: Database and Search Strategy

| Database | Search Strategy |
| --- | --- |
| Pubmed | ((((((cárie radicular [Termos MeSH]) OR (cárie radicular)) OR (cárie, raiz)) OR (cárie cervical)) OR (cárie cervical)) OR (cárie cervical)) OU ( Cárie cervical)  ((((((((((((((((() (((((((((()) (((((((((((((((() ((((((()) (Doenças, Periodontal)) OU (Doença Periodontal)) OU (Paradontose)) OU (Paradontose)) OU (Piorreia Alveolar)) OU (Doenças gengivais [Termos MeSH])) OU (Doenças gengivais)) OU (Doença, gengival) ) OU (doenças,Gengival)) OR (doença gengival)) OR (epúlides)) OR (epúlide)) OR (gengivose)) OR (gengivoses)) OR (atrofia periodontal [termos MeSH])) OR (atrofia periodontal)) OR (atrofia periodontal) ) OU (atrofia do periodonto) OU (atrofia do periodonto)) OU (atrofia do periodonto) OU (atrofia gengivo-óssea)) OU (atrofia gengivo-óssea)) OU (atrofia gengivo-óssea)) OU (perda óssea alveolar [MeSH Termos])) OU (perda óssea alveolar)) OU (perdas ósseas alveolares)) OU (atrofia do processo alveolar)) OU (atrofia do processo alveolar)) OU (reabsorção alveolar)) OU (reabsorção alveolar)) OU (reabsorção alveolar) ) OU (reabsorções, alveolar) OU (perda óssea, periodontal)) OU (perdas ósseas, periodontais)) OU (perdas ósseas periodontais)) OU (perda óssea periodontal)) OU (reabsorção periodontal)) OU (reabsorções periodontais)) OU (reabsorção,Periodontal)) OU (atrofia óssea alveolar)) OU (atrofia óssea alveolar)) OU (atrofia óssea alveolar)) OU (atrofia óssea alveolar)) OU (perda óssea alveolar)) OU (periodontite [termos MeSH])) OU (Periodontite)) OU (Periodontite)) OU (Pericementite)) OU (Pericementites)) OU (Periodontite Agressiva [Termos MeSH])) OU (Periodontite Agressiva)) OU (Periodontite Agressiva)) OU (Periodontite Pré-púbere)) OU (Periodontite Circumpubertal) OU (Periodontite Circumpubertal)) OU (Periodontite Pré-púbere)) OU (Periodontite de início precoce)) OU (Periodontite de início precoce)) OU (Periodontites de início precoce)) OU (Periodontites de início precoce) ) OU (Periodontite, Início Precoce)) OU (Periodontite Juvenil)) OU (Periodontite Juvenil)) OU (Periodontites Juvenis)) OU (Periodontites,Juvenil)) OU (Periodontose)) OU (Periodontoses)) OU (Periodontite, Agressiva, 1)) OU (Periodontite Crônica [Termos MeSH])) OU (Periodontite Crônica)) OU (Periodontites Crônicas)) OU (Periodontites Crônicas) ) OU (Periodontite Crônica)) OU (Periodontite em Adultos)) OU (Periodontites em Adultos)) OU (Periodontites em Adultos)) OU (Periodontites em Adultos)  (((((((((Humanos [Termos MeSH]) OR (Humanos)) OR (Homo sapiens)) OR (Homem (Taxonomia))) OR (Homem, Moderno)) OU (Homem Moderno)) OU (Humano)) OU (Adulto [Termos MeSH])) OU (Adulto)) OU (Adultos)  #1 AND #2 AND #3 |
|  |  |
| Scopus | ( TITLE-ABS-KEY ( human* ) OR TITLE-ABS-KEY ( "Modern Man" ) OR TITLE-ABS-KEY ( "Man (Taxonomy)" ) OR TITLE-ABS-KEY ( "Homo sapiens") AND TITLE-ABS-KEY ( adult* ) ) AND ( TITLE-ABS-KEY ( "Periodontal Disease*" ) OR TITLE-ABS-KEY ( "Parodontos*" ) OR TITLE-ABS-KEY ( "Pyorrhea Alveolaris" ) OR TITLE-ABS-KEY ( "Gingival Disease*" ) OR TITLE-ABS-KEY ( epuli* ) OR TITLE-ABS-KEY ( gingivos* ) OR TITLE-ABS-KEY ( "Periodontal Atroph*" ) OR TITLE-ABS-KEY ( "Atrophy of Periodontium" ) OR TITLE-ABS-KEY ( "Periodontium Atroph*" ) OR TITLE-ABS-KEY ( "Gingivo-Osseous Atroph*" ) OR TITLE-ABS-KEY ( "Gingivo Osseous Atroph*" ) OR TITLE-ABS-KEY ( "Alveolar Bone Loss*" ) OR TITLE-ABS-KEY ( "Alveolar Process Atroph*" ) OR TITLE-ABS-KEY ( "Alveolar Resorption*" ) OR TITLE-ABS-KEY ( "Periodontal Bone Loss*" ) OR TITLE-ABS-KEY ( "Periodontal Resorption*" ) OR TITLE-ABS-KEY ( "Alveolar Bone Atroph*" ) OR TITLE-ABS-KEY ( periodontiti* ) OR TITLE-ABS-KEY ( "Circumpubertal Periodonti*" ) OR TITLE-ABS-KEY ( "Prepubertal Periodontitis" ) OR TITLE-ABS-KEY ( "Early-Onset Periodonti*" ) OR TITLE-ABS-KEY ( "Early Onset Periodonti*" ) OR TITLE-ABS-KEY ( "Juvenile Periodontiti*" ) OR TITLE-ABS-KEY ( periodontos* ) OR TITLE-ABS-KEY ( "Chronic Periodontiti*" ) OR TITLE-ABS-KEY ( "Adult Periodontiti*" ) ) AND ( TITLE-ABS-KEY ( "Root Caries " ) OR TITLE-ABS-KEY ( "Cervical Car*" ) |
| Web of Science | TS= (human*) OR TS= ("Modern Man") OR TS= ("Man (Taxonomy)") OR TS= ("Homo sapiens") AND TS= (adult*)  TS= (“Periodontal Disease*”) OR TS= (“Parodontos*”) OR TS= ("Pyorrhea Alveolaris") OR TS= ("Gingival Disease*") OR TS= (Epuli*) OR TS= (Gingivos*) OR TS= ("Periodontal Atroph*”) OR TS= ("Atrophy of Periodontium") OR TS= ("Periodontium Atroph*") OR TS= ("Gingivo-Osseous Atroph*") OR TS= ("Gingivo Osseous Atroph*") OR TS= ("Alveolar Bone Loss*") OR TS= ("Alveolar Process Atroph*") OR TS= ("Alveolar Resorption*") OR TS= ("Periodontal Bone Loss*") OR TS= ("Periodontal Resorption*") OR TS= ("Alveolar Bone Atroph*") OR TS= (Periodontiti*) OR TS= ("Circumpubertal Periodonti*") OR TS= ("Prepubertal Periodontitis") OR TS= ("Early-Onset Periodonti*”) OR TS= ("Early Onset Periodonti*") OR TS= ("Juvenile Periodontiti*") OR TS= (Periodontos*) OR TS= ("Chronic Periodontiti*”) OR TS= ("Adult Periodontiti*") OR TS=(“Gingival Disease*”) OR TS=(Gingiviti*) OR TS= (“Gum disease*”)  TS= ("Root Car*") OR TS= ("Cervical Car*") OR TS= (“Caries of the root”) OR TS= (“dental root”)  #1 AND #2 AND #3 |
|  |  |
| The Cochrane Library | (human*) OR ("Modern Man") OR ("Man (Taxonomy)") OR ("Homo sapiens") AND (adult*)  (“Periodontal Disease*”) OR (“Parodontos*”) OR ("Pyorrhea Alveolaris") OR ("Gingival Disease*") OR (Epuli*) OR (Gingivos*) OR ("Periodontal Atroph*”) OR ("Atrophy of Periodontium") OR ("Periodontium Atroph*") OR ("Gingivo-Osseous Atroph*") OR ("Gingivo Osseous Atroph*") OR ("Alveolar Bone Loss*") OR ("Alveolar Process Atroph*") OR ("Alveolar Resorption*") OR ("Periodontal Bone Loss*") OR ("Periodontal Resorption*") OR ("Alveolar Bone Atroph*") OR (Periodontiti*) OR ("Circumpubertal Periodonti*") OR ("Prepubertal Periodontitis") OR ("Early-Onset Periodonti*”) OR ("Early Onset Periodonti*") OR ("Juvenile Periodontiti*") OR (Periodontos*) OR ("Chronic Periodontiti*”) OR ("Adult Periodontiti*") OR (“Gingival Disease*”) OR (Gingiviti*) OR (“Gum disease*”)  ("Root Car*") OR ("Cervical Car*") OR (“Caries of the root”) OR (“dental root”)  #1 AND #2 AND #3 |
| LILACS | (human$ OR (Modern Man) OR (Man (Taxonomy)) OR (Homo sapiens) AND adult$ ) AND ((Periodontal Disease$) OR Parodontos$ OR (Pyorrhea Alveolaris) OR (Gingival Disease$) OR Epuli$ OR Gingivos$ OR (Periodontal Atroph$) OR (Atrophy of Periodontium) OR (Periodontium Atroph$) OR (Gingivo-Osseous Atroph$) OR (Gingivo Osseous Atroph$) OR (Alveolar Bone Loss$) OR (Alveolar Process Atroph$) OR (Alveolar Resorption$) OR (Periodontal Bone Loss$) OR (Periodontal Resorption$) OR (Alveolar Bone Atroph$) OR Periodontiti$ OR (Circumpubertal Periodonti$) OR (Prepubertal Periodontitis) OR (Early-Onset Periodonti$) OR (Early Onset Periodonti$) OR (Juvenile Periodontiti$) OR Periodontos$ OR (Chronic Periodontiti$) OR (Adult Periodontiti$)) AND ((Root Car$) OR (Cervical Car$) ) |
| OpenGrey | Human AND “periodontal disease” AND “root caries” |
| Google Scholar | Human + Adults + “periodontal disease” + “root caries”– review – animal –children-"in vitro"- book |

Supplementary Table 2. Description of confounding domains identified in selected studies.

|  | **Confounding domain** | **Description** | **Examples identified in selected studies** |
| --- | --- | --- | --- |
| 1 | Sociodemographic and socioeconomic | Sociodemographic and socioeconomic characteristics. | Age; Area of residence (degree of urbanization); Area of residence (urban or rural); Country; Education level; Ethnicity; Household income per capita; Job classification; Local of residence (province, municipality, and autonomous region); Proportion of population employed in agriculture; Proportion of population employed in industry; Sex; Social economic status; Social insurance region. |
| 2 | Behaviors | Behaviors and daily habits. | Alcohol consumption; Frequency of dental visits; Frequency of eating between meals; Frequency of sugar consumption; Frequency of toothbrushing; Smoking status; Use of a toothpick; Use of dental floss. |
| 3 | Dental | Dental characteristics clinically assessed. | DFT (type of caries by mean of affected surface); DMFT; Number of root surface decay; Number of teeth; Root caries; Root surface decay incidence; Root surface decayed and filled teeth; Root surface decayed teeth; Root surface filled teeth. |
| 4 | Periodontal | Periodontal characteristics clinically assessed. | Attachment loss; Gingival inflammation; Healthy periodontal status; Periodontitis (degree by clinical attachment loss); Periodontitis (diagnosis by pocket probing depth and attachment loss); Pocket probing depth; Subgingival plaque retention. |
| 5 | Microbiological | Microbiological parameters assessed by sample test. | Level of bacteria (Lactobacilli); Level of bacteria (Streptococcus mutans). |
| 6 | Salivary | Salivary parameters clinically assessed. | Stimulated saliva flow. |
| 7 | Comorbidities | General disease possibly associated with the study outcome. | Diabetes. |

DFT - Decayed and Filled Teeth; DMFT - Decayed, Missing due to caries, and Filled Teeth.
